# Membrane TNF confers protection to acute mycobacterial infection

**DOI:** 10.1186/1465-9921-6-136

**Published:** 2005-11-14

**Authors:** Cecile Fremond, Nasiema Allie, Ivy Dambuza, Sergei I Grivennikov, Vladimir Yeremeev, Valerie FJ Quesniaux, Muazzam Jacobs, Bernhard Ryffel

**Affiliations:** 1 Molecular Immunology and Embryology, Centre National de la Recherche Scientifique, Orléans, France; 2 Department of Immunology, Institute of Infectious Disease and Molecular Medicine, University of Cape Town, South Africa; 3 Laboratory of Molecular Immunoregulation, Center for Cancer Research, National Cancer Institute-Frederick, Fort-Detrick, Frederick, MD 21702, USA; 4 Max Planck Institute for Infection Biology, Department of Immunology, Schumannstrabe 21/22, 10117 Berlin, Germany

**Keywords:** Mycobacterium tuberculosis H37Rv, membrane TNF, TNF-deficiency, T cell recruitment, granuloma

## Abstract

**Background:**

Tumour necrosis factor (TNF) is crucial for the control of mycobacterial infection as TNF deficient (KO) die rapidly of uncontrolled infection with necrotic pneumonia. Here we investigated the role of membrane TNF for host resistance in knock-in mice with a non-cleavable and regulated allele (mem-TNF).

**Methods:**

C57BL/6, TNF KO and mem-TNF mice were infected with *M. tuberculosis *H37Rv (*Mtb *at 100 CFU by intranasal administration) and the survival, bacterial load, lung pathology and immunological parameters were investigated. Bone marrow and lymphocytes transfers were used to test the role of membrane TNF to confer resistance to TNF KO mice.

**Results:**

While TNF-KO mice succumbed to infection within 4–5 weeks, mem-TNF mice recruited normally T cells and macrophages, developed mature granuloma in the lung and controlled acute *Mtb *infection. However, during the chronic phase of infection mem-TNF mice succumbed to disseminated infection with necrotic pneumonia at about 150 days. Reconstitution of irradiated TNF-KO mice with mem-TNF derived bone marrow cells, but not with lymphocytes, conferred host resistance to *Mtb *infection in TNF-KO mice.

**Conclusion:**

Membrane expressed TNF is sufficient to allow cell-cell signalling and control of acute *Mtb *infection. Bone marrow cells, but not lymphocytes from mem-TNF mice confer resistance to infection in TNF-KO mice. Long-term infection control with chronic inflammation likely disrupting TNF mediated cell-cell signalling, additionally requires soluble TNF.

## Background

Protective immunity to *M. tuberculosis *(*Mtb*) infection is regulated by T cells, macrophages and cytokines including IFNγ, IL-12 and TNF [1,2]. IFNγ derived from T and NK cells has been shown to be essential, as mice with a disruption of the IFNγ signalling are unable to restrict the growth of *M. tuberculosis *and succumb to the infection [3-6]. A critical role for TNF in mycobacterial defence was inferred from neutralisation and gene deletion experiments in mice [7-10]. TNF neutralising therapies for rheumatoid arthritis and Crohn's disease turned out to increase the risk of developing tuberculosis (TB) and other opportunistic infections [11-14].

TNF produced by macrophages, and a variety of other cells, is a major regulator of inflammation and leukocyte trafficking [15,16]. Although soluble TNF in controlling intracellular bacterial infections is uncontested, the function of membrane TNF, which is subsequently cleaved by the metalloproteinase-disintegrin TACE (TNFα converting enzyme) [17] into the secreted trimeric TNF, is not established. Several biological functions of membrane TNF have been described, such as strong cytotoxity, polyclonal activation of B cells, induction of IL-10 by monocytes, ICAM-1 expression on endothelial cells and regulation of chemokine expression [18,19]. The transgenic overexpression of membrane TNF (mem-TNF) demonstrated an *in vivo *role in the control of Listeria and mycobacterial infection [20,21]. However these studies have been performed on TNF/lymphotoxin (LT) deficient background, and since lymphotoxin is implicated into TB resistance, do not address the role of membrane TNF only [20,21]. The recent generation of a mouse with functional, normally regulated and expressed membrane-bound TNF, obtained by knocking-in an uncleavable Δ1–9,K11E TNF allele, represents a major advance and allowed interesting insights in the role of membrane TNF in lymphoid structure maintenance and inflammation [19].

Here we asked whether membrane TNF is sufficient for containing *Mtb *infection. We compared the host resistance to acute mycobacterial infection in mem-TNF mice, eg. Δ1–9,K11E TNF knock-in mice [19], and TNF-deficient (TNF-KO) mice [22]. We show that membrane TNF substitutes soluble TNF to recruit and activate macrophages and T cells, to generate granuloma and control acute infection, but is insufficient to control the chronic phase of infection. Transfer of bone marrow cells, but not of lymphocytes from mem-TNF and WT mice was able to confer resistance to infection in TNF KO mice.

## Methods

### Mice

Mem-TNF [19] and TNF-KO mice [22] on a C57BL/6 background and C57BL/6 mice were bred in house. For experiments, adult (8–15 week old) animals were kept in sterile isolators in a biohazard animal unit. All animal experiments complied with the French Government's ethical and animal experiment regulations.

### Bacteria and infection

Pulmonary infection with *M. tuberculosis *H37Rv (Pasteur) was performed by delivering 100 bacteria into both nasal cavities (20 μl each) under xylazine-ketamine anaesthesia as described [23]. The bacterial load in the lung was determined at day 1 post infection. Three independent experiments were conducted, one short term for 90 days, a long-term study over 240 days (n = 6 per group) and a second long-term study over 200 days (n = 10 mice per group).

### Bacterial load in tissues and histological investigation

Bacterial loads in organs of infected mice were evaluated at different time points after infection with *M. tuberculosis *H37Rv as described [23]. For histological analysis lungs were fixed in 4% phosphate buffered formalin, paraffin-embedded as described, and stained with haematoxylin and eosin and a modified Ziehl-Neelsen [10].

### FACS analysis of infiltrating cells from infected lung

FACS analysis of inflammatory cells from infected lung was performed as described [23,24]. Rat anti-mouse CD4-PerCP (clone RM4-5), CD8-FITC (clone 53-6.7), Ly6G-PE (clone RD6-8C5), CD11b-PE (clone M1/70), I-A/I-E- FITC (clone 2G9) were from BD Pharmingen (San Diego, CA) and stained cells were analyzed by flow cytometry on a LSR analyser (Becton Dickinson).

### Cytokine determination

IL-12p40 and IFNγ were quantified using commercial ELISA (Duoset, R&D Systems, Abingdon, UK). Bioactive TNF was assessed using the WEHI 164 cells based bioassay [25].

### Reconstitution of irradiated mice with lymphocytes or bone marrow cells of TNF KO mice

Haemopoietic reconstitution of bone marrow was performed as described [26]. Briefly, designated recipient mice received an optimised lethal total body irradiation dose of 8 Grey using a γ-irradiation source (CHRO, Orleans). Irradiated mice were reconstituted with 2 × 10^6 ^fresh unseparated bone marrow cells by intravenous injection in the lateral tail vein (n = 6 per group). Mice were left to fully reconstitute for at least three month prior to infection, reconstitution was verified by the analysis of the haematogram. In addition, freshly isolated splenic T lymphocytes (10^7 ^i.v.) from WT and mem-TNF mice were transferred into TNF KO mice (exposed to 4 Grey γ-irradiation) immediately before *Mtb *infection and survival was followed (n = 6 per group). The experiments were repeated once.

### Primary macrophage and dendritic cells cultures

Murine bone marrow cells were isolated from femurs and differentiated into macrophages using 20% horse serum and 30% L929 cell-conditioned medium as a source of M-CSF [26]. Alternatively, murine bone marrow cells were differentiated into myeloid dendritic cells using 4% J558L cell-conditioned medium as a source of GM-CSF [27].

### Stimulation of macrophages and dendritic cells

Bone marrow derived macrophages (BMDM) and dendritic cells (BMDC) were plated (at 10^5 ^cells/well) and stimulated with LPS (*Escherichia coli*, serotype O111:B4, Sigma, St Louis, MO, at 100 ng/ml) or *M. tuberculosis *H37Rv (heat-killed 40 min at 80°C; 2 bacteria per cell), or infected with *M*. *bovis *BCG (Pasteur Institute, at a MOI of 2 bacteria per cell) as described before [28]. Cell supernatants were harvested after 24 h of stimulation in the presence of IFNγ (100 U/ml) for TNF and IL-12 p40 quantification, and nitrite measurements by Griess reagents [29]. Membrane expressed CD40 and CD86 staining was performed as described [28] using CD40-PE (clone 3/23) and CD86-FITC (clone GL1, BD PharMingen San Diego, CA). The mean fluorescence of non-stimulated and activated macrophages was compared.

### Antigen-specific IFNγ production

T cell priming was assessed by the production of IFNγ upon antigen restimulation ex vivo as described [23]. Single cell suspension of splenocytes and mediastinal lymph nodes were prepared from mice 4 weeks after infection. Cells were stimulated with either 2.5 μg/ml Con A (Sigma), a lyophilised soluble fraction from BCG culture supernatant (SupBCG, 10 μg/ml), or heat killed Listeria monocytogenes (100 bacteria per cell) for 3 days at 37°C as described [28]. IFNγ production in the supernatant was quantified by ELISA.

### Statistical Analysis

ANOVA was used for the analysis of the early data points for the comparison of three experimental groups. For the data points beyond one month we used the Student's t test. Survival data (Kaplan Meier plots) were compared using a log rank test for the comparison of the groups. A p value <0.05 was considered statistically significant.

## Results

### Absence of secreted TNF in mem-TNF mice

**Figure 1:**
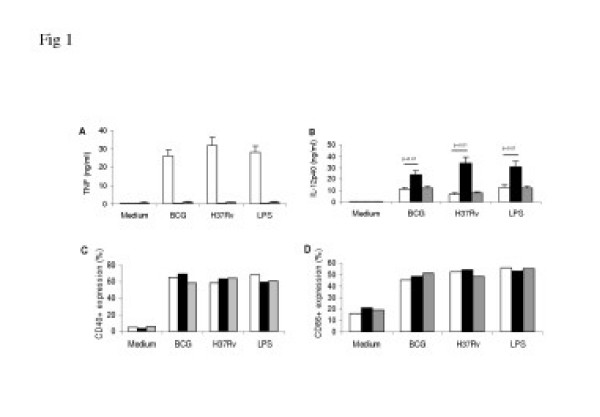
**Defective soluble TNF and augmented IL-12 production from mycobacterial stimulated macrophages, and normal upregulation of CD40 and CD86 costimulatory molecules. **Mycobacteria induced TNF (A) and IL-12p40 (B) production at 24 h TNF in the supernatant of macrophages from WT (open bars), TNF-deficient (black bars) and mem-TNF mice (grey bars) infected with BCG or H37Rv (at a MOI of 2) or stimulated with LPS (100 ng/ml). Data are expressed as the mean ± SD (n = 2 mice from one out of 4 independent experiments). TNF independent expression of costimulatory molecules CD40 (C) and CD86 (D) analysed by flow cytometry in macrophages stimulated as above. Results are expressed as mean percentage.

To confirm that mem-TNF mice do not secrete TNF, serum was obtained 90 min after LPS (100 μg) injection. In contrast to WT mice TNF was undetectable in the sera of both TNF-KO and mem-TNF mice (not shown). While bone marrow derived macrophages (BMDM) secrete high TNF levels in response to *M. bovis *BCG and *Mtb *infection or to LPS, TNF is undetectable in culture supernatants of BMDM from TNF-KO and strongly defective in mem-TNF mice (Fig 1A), the latter however express TNF on the membrane of activated macrophages and T-cells as shown before [19]. Interestingly, the production of IL-12p40 was augmented in TNF-KO as compared to WT macrophage, but normalised in mem-TNF macrophages (Fig 1B). Therefore, membrane-bound TNF is sufficient to normalise IL12p40 production by macrophages, while complete absence of TNF results in deregulated IL12p40 expression as described before [30]. The production of IL-6 and nitrites was essentially unaffected in stimulated mem-TNF macrophages, as compared to WT or TNF-KO macrophages (data not shown). Second, mycobacteria-induced expression of costimulatory molecules CD40 and CD86 was investigated in BMDM and BMDC. CD40 and CD86 upregulation was induced to a comparable extent in macrophages (Fig 1C,D) and dendritic cells (data not shown) in all groups as compared to unstimulated controls indicating that the expression of costimulatory molecules is TNF independent. Taken together, these observations confirm the defective secretion of functional TNF *in vivo *and *in vitro *in mem-TNF mice, while the expression IL-12 and costimulatory molecules were unaffected.

### Membrane TNF is sufficient to control acute M. tuberculosis infection

**Figure 2:**
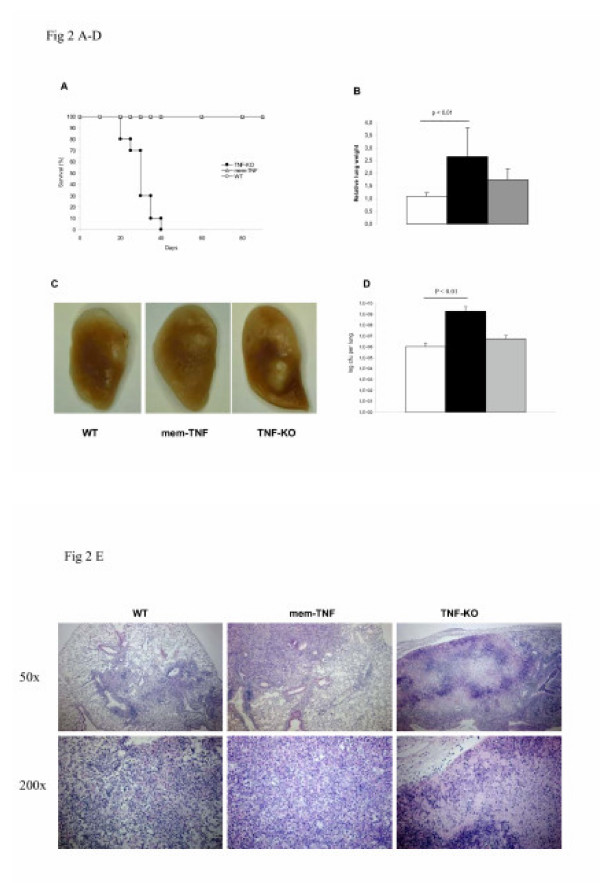
**Necrotic pneumonia and uncontrolled infection in TNF-KO mice, but not in mem-TNF mice at 30 days after *Mtb *infection (100 CFU, intranasal administration). **Survival (A), relative lung weights (B), macroscopic lung changes (C), and bacterial load at 30 days in the lungs (D). The results are expressed as the mean ± SD (n = 6 per group) and are representative of two independent experiments. The bacterial load at day 1 upon infection was 84 ± 28 CFU per lung (n = 6). Microscopic investigations of the lungs show confluent neutrophil and mononuclear cell inflammation with extensive, confluent necrosis and abundant bacilli in TNF-KO mice, while mem-TNF and WT mice show focal, largely perivascular mononuclear cell infiltration (E). Representative haematoxylin and eosin stained lung sections at low (50×) and high power (200×) are shown (n = 5 per group).

To ascertain the role of soluble vs membrane-bound TNF in resistance to tuberculosis, we compared mem-TNF, TNF-KO mice and WT mice infected with 100 CFU *Mtb *given by intranasal administration. Within 4–5 weeks of infection TNF-KO mice displayed rapid weight loss (not shown), impeded locomotor activity and succumbed to infection rapidly (Fig 2A). Mem-TNF mice had normal weight development and survived the 90 days observation period. Therefore, we conclude that membrane bound TNF confers substantial protection to acute *Mtb *infection.

The relative lung weights -a surrogate marker of inflammation- of surviving TNF-KO mice at 30 days were increased (p < 0.01), but not in WT and only slightly in mem-TNF mice (Fig 2B). Lungs of TNF-KO mice displayed large nodular and confluent lesions on the pleura at 30 days post infection, which were much smaller and more discrete in mem-TNF mice, comparable to those in WT mice (Fig. 2C).

We then determined the mycobacterial load in the lungs. At 30 days WT mice displayed a 4 log increase of viable mycobacteria reaching 10^6 ^CFU counts in the lungs (Fig 2D), which stabilises thereafter, as shown before [24]. By contrast, bacillary burden in the lung was significantly increased in TNF-KO mice (p < 0.01) reaching a value of 10^9 ^CFU, while mem-TNF had comparable CFU values as WT mice (Fig 2D). *Mtb *dissemination measured by splenic and hepatic bacterial loads was also significantly increased in TNF-KO mice, but not in mem-TNF mice (data not shown). Therefore, acute *Mtb *infection is controlled in the presence of membrane TNF only.

The establishment of granulomas is the manifestation of a vigorous cell mediated immune response, which is crucial for inhibiting mycobacterial growth and depends on TNF [31]. We asked whether membrane bound TNF is sufficient for granuloma formation upon *Mtb *infection. At 30 days post infection the lungs of mem-TNF mice displayed well-defined granulomatous lesions that were characterised by foamy epitheloid like macrophages with surrounding and interspersed and perivascular lymphocytic infiltration, similar to the granuloma structures formed in WT mice (Fig 2E). TNF-KO mice had abundant inflammatory cells with extensive necrosis, and no structured granuloma. Therefore, membrane TNF is sufficient for granuloma formation and infection control.

### Membrane TNF allows lymphocyte recruitment and cell activation upon mycobacterial infection

**Figure 3:**
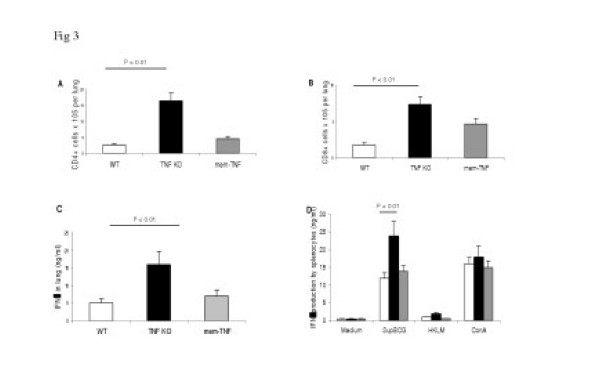
**Augmented CD4 and CD8 T cell recruitment, pulmonary IFNγ production and antigen-specific T cell response in infected TNF-KO mice are corrected in mem-TNF mice. **Recruitment of CD4 (A) and CD8 T cells (B) in the lung of WT, mem-TNF and TNF-KO mice infected with *Mtb *(100 CFU). Lymphocytes were obtained form lungs from *Mtb *infected mice at 30 days post infection as described in Materials and Methods. IFNγ levels in lung homogenates at 30 days of infection (C): Increased IFNγ levels in TNF KO mice, which were lower and comparable in mem-TNF and WT mice. Antigen-specific IFNγ production by SupBCG restimulated splenocytes from mem-TNF and TNF-KO mice, but augmented response in TNF-KO splenocytes (D). Data are representative of two independent experiment (n = 4 mice, mean ± SD).

In view of the granulomatous response elicited in mem-TNF mice after *Mtb *infection we investigated whether membrane TNF could affect the cellular recruitment in the lungs. Single cell suspension of lung infiltrating cells was obtained at 30 days post infection. CD4 and CD8 lymphocytes were significantly higher in TNF-KO than in WT mice (p < 0.01), in line with the severe pathology in these mice, while the sole presence of mem-TNF prevented the augmented lymphocyte recruitment as shown in mem-TNF mice (Fig. 3A,B). Enhanced cell mediated immune responses have been shown in the complete absence of TNF [32] and is probably linked to uncontrolled bacterial growth in TNF-KO mice, leading to excessive inflammation. We show that the presence of membrane TNF corrects the TNF-deficient phenotype, i.e. cellular recruitment to the infected lungs was normal. Concomitant with increased lymphocyte recruitment IFNγ levels in lung homogenates from TNF-KO mice were significantly increased (p < 0.01) as compared to WT mice (Fig. 3C). By contrast, lungs from mem-TNF mice showed comparable amounts of IFNγ as WT mice.

We then asked whether a mycobacteria-specific T cell response is acquired in mem-TNF and TNF-KO mice. Ex vivo restimulation 30 days after infection of splenic lymphocytes (Fig. 3D) with mycobacterial antigens (SupBCG), but not with an irrelevant antigen, induced IFNγ secretion in cells from both TNF-KO and mem-TNF mice. Although IFNγ levels were significantly higher in TNF-KO as compared with WT (p < 0.01), the IFNγ levels of mem-TNF mice did not differ from those of infected WT mice.

These data suggest that membrane TNF allows substantial, but controlled recruitment and activation of T cells and macrophages resulting in mycobactericidal effector mechanisms. The acquisition of an antigen specific immune response as assessed by the production of IFNγ is TNF independent.

### Membrane TNF is unable to confer long-term protection against Mtb infection

**Figure 4:**
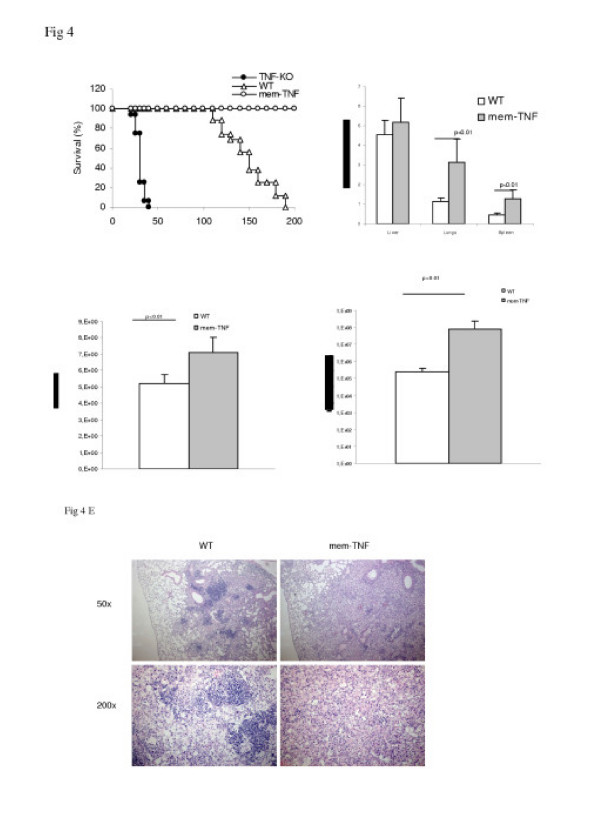
**Mem-TNF mice succumb of uncontrolled chronic *Mtb *infection after 4–6 months. **Survival (A) of infected mice (*Mtb *100 CFU given by intranasal route), data two independent experiments (n = 16 per group). Organs weights (B) and bacterial loads (CFU) in lungs (C) and spleen (D) were determined at 112 days after infection. The results are expressed as the mean ± SD (n = 6 per group). Microscopic changes of the lung from mem-TNF and WT mice at 112 days (E). Microscopic analysis of the lung reveal a more abundant macrophages and lymphocyte infiltration in mem-TNF mice with confluent foci and less defined granulomatous lesions. Representative haematoxylin and eosin stained lung sections at low (50×) and high power (200×) are shown (n = 6 per group).

While acute infection was controlled by the expression of membrane TNF (Fig. 2), absence of soluble TNF might affect the control of chronic infection, as we have shown in other conditions [24]. Conversely, in TLR2 [33] or TLR4 deficient mice [34] acute, but not chronic infection is controlled. We therefore conducted a long-term infection study in mem-TNF mice and compared it to WT mice infected with 100 CFU (intranasal route), a condition where TNF-KO mice die within 4–5 weeks (Fig. 2A) [10,23]. While mem-TNF mice appeared healthy over four months, they started to loose body weight and succumbed to infection between 130 – 170 days (Fig. 4A). At 112 days, the relative lung and spleen weights as indicator of inflammation and pathology were significantly increased in mem-TNF mice (Fig. 4B) and viable mycobacteria in lungs were almost two logs higher (Fig. 4C), reaching 10^7 ^CFU, a bacterial load still much lower than that observed in moribund TNF-KO mice after 30 days of infection (Fig. 2D). Bacterial dissemination was also increased (Fig. 4D). Mycobacterial cultures obtained from lungs from moribund mem-TNF mice sacrificed at later time points indicated a further increase of CFU in the lungs (not shown). The microscopic investigation revealed a more abundant macrophages and lymphocyte infiltration in mem-TNF mice with confluent foci and less defined granulomatous lesions at 112 days (Fig. 4E). Therefore, lack of soluble TNF may allow slow growth of mycobacteria during the chronic infection, with likely progressive hypoxemia due to chronic pneumonia, leading to death in the chronic phase of infection.

### Reconstitution of TNF deficiency by bone marrow transplantation

First we attempted to confer resistance in irradiated TNF KO mice to acute *Mtb *infection by the transfer of lymphocytes from mem-TNF mice. Neither lymphocytes from mem-TNF nor WT mice increased survival in TNF KO mice (data not shown), which is at variance with the recent data from Saunders et al. [37].

**Figure 5:**
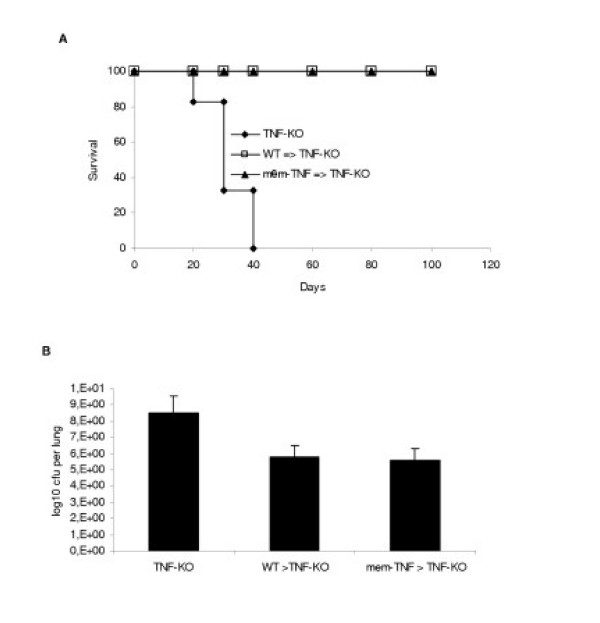
**Bone marrow cells from mem-TNF mice correct the heightened susceptibility of TNF deficient mice. **Long-term survival of TNF-KO mice reconstituted with bone marrow from mem-TNF or WT mice (A), and control of infection in the lung (B) of bone marrow reconstituted TNF-KO mice (p < 0.01). Lethally irradiated (8 Grey) and reconstituted TNF-KO were exposed to infection (*Mtb *100 CFU, intranasal administration) 4 months after irradiation and bone marrow reconstitution. The results are expressed as mean values ± SD (n = 6).

Since TNF derived from hemopoietic cells likely contributes most of bioactive TNF [35] and we have shown before that bone marrow transplantation confers resistance to TNF-KO mice to BCG infection [36], we asked whether membrane expressed TNF on haemopoietic cells might be sufficient to correct the susceptibility to *Mtb *infection of TNF-KO mice. Lethally irradiated TNF-KO mice reconstituted with bone marrow cells from mem-TNF mice controlled *Mtb *infection as demonstrated by survival over three months, while TNF-KO mice succumbed to acute necrotic pneumonia within 40 days (Fig. 5A). Similarly, irradiated TNF-KO reconstituted with WT bone marrow survived the whole experiment. Although TNF-KO mice could not contain the infection, reconstituted TNF-KO mice developed granuloma (data not shown) and were able to control acute infection and bacterial growth in the lungs (Fig. 5B). Therefore, bone marrow derived cells expressing membrane TNF, but not lymphocytes, are sufficient to control infection in reconstituted TNF-KO mice.

## Discussion

We report that membrane TNF plays a crucial role in the control of mycobacterial infection using a knock-in mouse model where the endogenous TNF allele was replaced by a non-cleavable membrane TNF (mem-TNF) mutated in the TACE cleavage site [19]. The expression of mutated membrane TNF confers protection against acute mycobacterial infection with initial control of mycobacterial growth and normal granuloma development in the lung. However, long-term control of infection appears to be dependent additionally on soluble TNF, as mem-TNF mice eventually succumb to chronic infection as shown by a recent contribution [37]. However, we show here significant differences such as uncontrolled infection in the late phase with disseminating infection and the capacity of membrane expressing cells from the bone marrow to confer long-term -and not transient- resistance to *Mtb *infection in TNF deficient recipient mice.

A critical role of TNF for the effective control and resolution of mycobacterial infection has been demonstrated previously [7,10,24], which is mediated by TNFR1 [9], rather than TNFR2 signalling [38]. TNF provided by recombinant BCG expressing TNF may reconstitute granuloma formation and host response in TNF-KO, but not in TNFR1-KO mice, demonstrating the critical role of TNF and TNFR1 signalling [39]. Overexpressed membrane TNF conferred partial resistance to Listeria and mycobacterial infection in transgenic mice on a TNF deficient [20] or on a TNF-LTα double deficient background [21,40]. However, transgenic expression of mem-TNF results in artificially high, non-regulated and non-selective expression of membrane TNF which may make conclusions on the physiological function difficult. While Saunders et al [37] using the same genetic model demonstrate death during the chronic phase, we show here that the bacterial load in lung and spleen is augmented significantly within 3 month of infection with disseminated infection (Fig 4C,D) and increases further in the mice dying with necrotic pneumonia.

The mechanism how membrane TNF confers protection may be due to cell-to-cell contacts of T cells, macrophages and other cells and needs further investigations. Several biological functions of membrane TNF have been reported previously and a preferential TNFR2 signalling has been suggested in vitro [18], while in vivo both TNFR1 and TNFR2 were reported to contribute to the signalling by membrane bound TNF [21,41-43]. Since TNFR1 is crucial for the resistance against TB infection, our study suggests memTNF -> TNFR1 signalling as important for host defence. Furthermore, membrane TNF has been shown to be involved in reverse (outside-to-inside) signalling. Upon ligation of its receptor mem-TNF expressing cells are activated to express E-selectin [44]. Thus, membrane TNF at least in T cells might function as a bipolar positive regulator of inflammation either transmitting signals as a ligand to target cells or receiving signals through membrane TNF itself into T cells.

Membrane TNF is sufficient for the development of granulomas. In the absence of TNF there was no formation of well-defined granulomas [7,36]. Neutralisation of TNF by antibodies, which likely affects also membrane TNF, leads to the disruption of established granulomas and uncontrolled infection [31]. Activated macrophages expressing iNOS in granuloma are critical for bacterial killing [45-47]. iNOS expression is reduced in lungs of infected TNF-KO mice as compared to mem-TNF and WT mice (data not shown), which has been confirmed [21,40]. Further, the transfer of bone marrow cells expressing mem-TNF is sufficient to correct the increased susceptibility of TNF-KO mice, which clearly points to hemopoietic cells being critically involved in host resistance, and corroborates our previous data using WT bone marrow cells to correct the TNF-KO phenotype [36]. By contrast, we were unable to confer host resistance to TNF KO mice by passive transfer of mature lymphocytes as reported recently [37]. Therefore, sustained membrane expressed TNF on myeloid and lymphoid cells may be necessary to control infection.

Interestingly, lymphocyte recruitment was lower and comparable in mem-TNF mice and WT mice (Fig. 3A,B) unlike in the absence of TNF. Augmented lymphocyte recruitment with expansion of activated CD4 and CD8 T cells in the complete absence of TNF has been reported before [32]. Since the expression of costimulatory molecules (Fig. 1C,D) was normal in the absence of TNF, we tested the adaptive immune response. Lymphocytes form infected mem-TNF mice had normal antigen-induced INF-γ response, comparable to that of WT mice (Fig. 3D), suggesting a normal adaptive immune response, while the response was augmented in TNF deficient T cells, in line with previous findings [32]. CD4+ T-cells are critical for cell mediated immunity [1], but not sufficient as shown before for MyD88 deficiency [23].

Finally, the question arises why mem-TNF mice succumb in the chronic phase of infection. It may be hypothesized that membrane expressed TNF activates macrophages in the absence of soluble TNF to a certain level, but the killing may not be as effective as in WT macrophages. Secreted TNF and hence distal signalling is likely required in the chronic phase where direct cell contact between T cells and macrophages may be more difficult to achieve in the chronic inflammatory and fibrotic tissue and therefore soluble TNF may be more efficient to maintain the activation of the innate host defence.

## Conclusion

Membrane expressed TNF allows cell-cell signalling and control of acute *Mtb *infection. Bone marrow cell reconstitution, but not lymphocyte transfer from mem-TNF mice confer resistance to infection in TNF-KO mice. Long-term infection control, however, with chronic inflammation disrupting TNF mediated cell-cell signalling, requires additionally soluble TNF.

The data are of clinical significance, as neutralising therapies used to treat patients with rheumatoid arthritis may reactivate latent TB infection [11]. TNF blockers have different capability to inactivate TNF, anti-TNF antibodies (infliximab, remicade) binding both soluble and membrane bound TNF, whereas soluble TNFR2 (etanercept) binding preferentially soluble TNF [14]. Therefore novel strategies to disrupt more selectively the TNF – TNF-R interaction sparing mem-TNF may be associated with less infectious complications.

## Abbreviations

*Mtb *M. tuberculosis,

CFU colony forming unit,

mem-TNF membrane bound TNF

## Competing interests

The author(s) declare that they have no competing interests.

## Authors' contributions

CF and NA were driving the project by designing the protocol and conducting the infectious protocol together with ID and MJ. SG was advising on the genetic mouse model, PCR control and cell transfer experiments. VQ with VY conducted the in vitro cell culture experiments with FACS analysis. BR with MJ was responsible for the overall design and control of the studies.
